# Circadian clocks and their integration with metabolic and reproductive systems: our current understanding and its application to the management of dairy cows

**DOI:** 10.1093/jas/skac233

**Published:** 2022-07-01

**Authors:** Theresa M Casey, Karen Plaut

**Affiliations:** Department of Animal Sciences, Purdue University, West Lafayette, IN 47907, USA; Department of Animal Sciences, Purdue University, West Lafayette, IN 47907, USA

**Keywords:** dairy cow, circadian clocks, lactation, mammary, metabolism, reproduction

## Abstract

The circadian system is an inbuilt timekeeping mechanism that tracks the 24-h day through the generation of circadian rhythms. Circadian rhythms enable animals to forecast and anticipate regular changes in their environment, and orchestrate biochemical, physiological and behavioral events so that the right process occurs at the right time. The 24 h rhythms generated by circadian clocks are integrated into homeostatic feedback loops and repair pathways. Metabolic and reproductive systems are highly integrated with the circadian timing system and demonstrate reciprocal regulation. Circadian clocks set the timing of circadian rhythms by gathering temporal information from external and internal signals to include light and nutrients. Exogenous and endogenous factors that function as inputs to the circadian clocks can disrupt their timing when applied at unusual and inappropriate times, and are referred to as chronodisruptors. Changes in the natural light-dark cycle perturb the circadian system. Other chronodisrupters include inappropriately timed food intake and physical activity and biological stress. Knowledge of the biology underlying circadian clock timing is critical to understanding how to maximize health and production efficiency of cattle. Here we review circadian clocks and their function in the regulation of metabolic and reproductive systems as well as the consequence of circadian disruption on mammary development and lactation with a particular focus on recent research findings from studies of dairy cows.

## Introduction

Many modern dairy production systems expose cows to 24 h of light, activity and feed availability. These management factors can impact the temporal organization of an animal’s physiology and affect their welfare and production efficiency. Timing of behavior and physiology underlies health and homeostasis (López-Otín and Kroemer, 2021). Central to establishing timing are circadian clocks that generate daily and seasonal rhythms of physiology and behavior. The 24 h rhythms generated by circadian clocks are integrated into homeostatic feedback loops and repair pathways. Circadian rhythms enable animals to forecast and anticipate regular changes in their environment, and orchestrate biochemical, physiological and behavioral events so that the right process occurs at the right time. Responsiveness of the master clock in the suprachiasmatic nuclei (SCN) to light and peripheral clocks to feeding time enables synchronization and adaptation to seasonal changes in day length and food availability. However, the plasticity of the circadian system makes it vulnerable to disruption. Exogenous and endogenous factors that function as inputs to the circadian clocks disrupt their timing and the order of physiologic functions when applied at unusual and inappropriate times, and are referred to as chronodisruptors (Erren and Reiter, 2009b). Changes in the natural light-dark cycle perturb the circadian system. Other chronodisrupters include inappropriately timed food intake and physical activity and biological stress (Erren and Reiter, 2009a, Evans and Anderson, 2018). Nutritional quality also plays a role in maintaining robustness of the circadian system.

Here we review circadian clocks and their function in the regulation of metabolic and reproductive systems as well as the consequence of circadian disruption on mammary development and lactation with a particular focus on recent research findings from studies of dairy cows. Cows like all mammals show seasonal and daily variations in the timing of physiology and behavior. Metabolic and reproductive status interact and affect these variations, and are realized in the daily and seasonal rhythms of milk yield and composition. Knowledge of the biology underlying circadian clock timing is critical to understanding how to maximize cow health and production efficiency.

## Circadian Clock Mechanism and Organization

The circadian system is an inbuilt timekeeping mechanism that tracks the 24-h day through the generation of circadian rhythms. Cellular clocks are located in virtually every cell of the body and can be viewed as having three components: (1) *input* (zeitgebers) and a way to receive temporal information (e.g., hormones binding to receptors), (2) the *core molecular clock* that generates circadian rhythms of gene expression, and (3) *output* or clock controlled genes (Albrecht, 2012).

**Figure 1.**
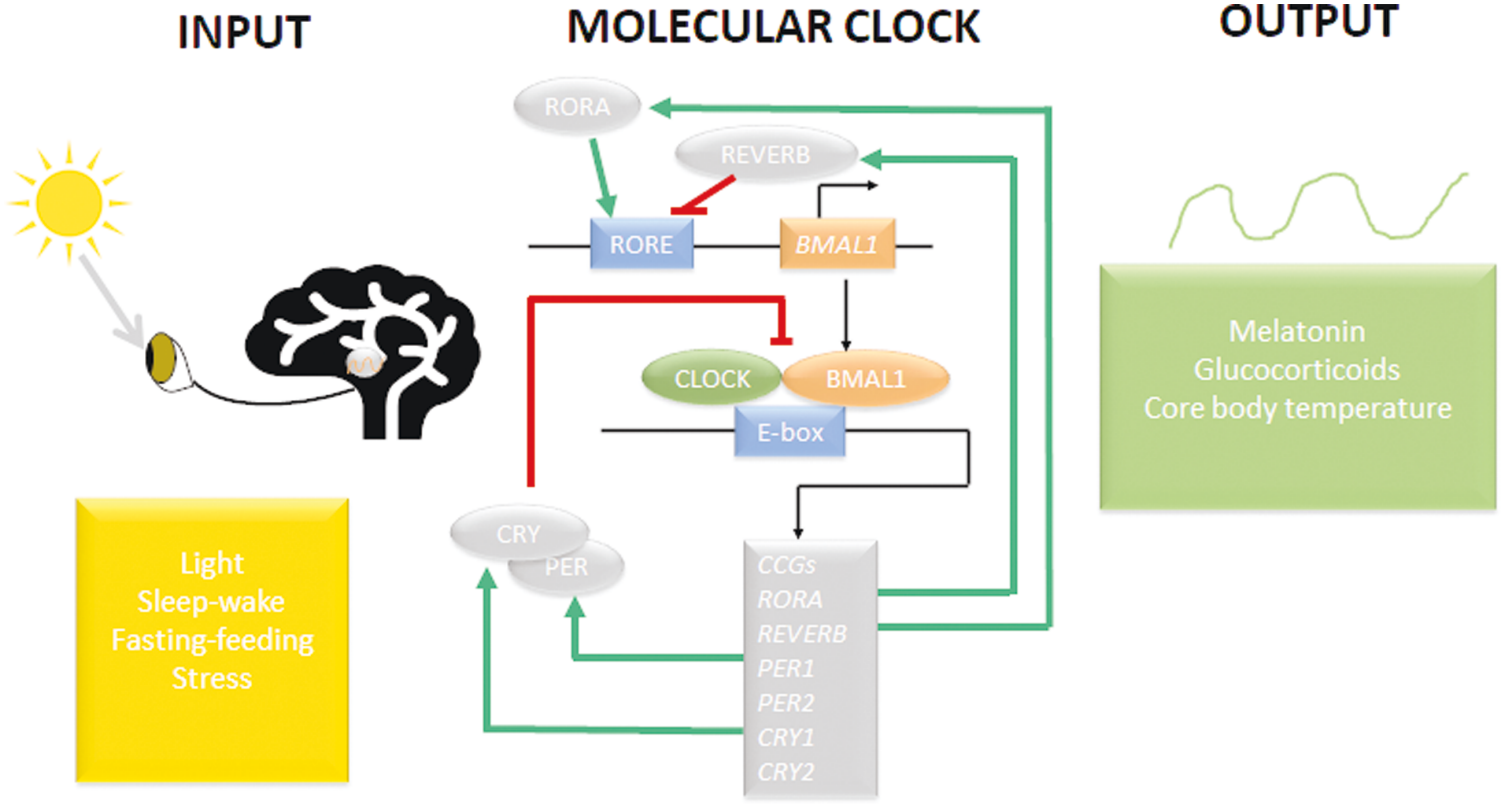
Schematic of the major inputs and outputs of the core circadian clock in the SCN. The core molecular clock is a transcription-translation autoregulatory feedback loop that generates circadian rhythms of clock-controlled genes (CCG). The positive loop consists of *BMAL1* and *CLOCK* gene products, and the negative loop consists of the *period* (*PER)* and *cryptochrome* (*CRY)* gene products. BMAL1 and CLOCK proteins heterodimerize and function as a transcription factor that binds the enhancer box (E-BOX) nucleotide sequence in the promoter region of clock—controlled genes (CCG), including their own repressors, PER and CRY, which form the transcription feedback loop. BMAL1 expression is also regulated by 2 its transcriptional targets, the nuclear receptors REV-ERBα and RORα, which, respectively, repress and active BMAL1 transcription by competing for the RORE promoter region, forming a secondary interlocked feedback loop.

The core molecular clock is a transcription-translation feedback loop of positive and negative elements (Fig. 1). *BMAL1* (aka *ARNTL*) and *CLOCK* are the positive elements of the feedback loop. The BMAL1:CLOCK heterodimer functions as a transcription factor that drives the expression of clock controlled genes, including their own negative regulators *Period* (*PER*) and *Cryptochrome* (*CRY*) genes. Upon translation PER and CRY proteins heterodimerize and prevent the binding activity of the BMAL1:CLOCK transcription factor to the enhancer box sequence (E-box, nucleotides CANNTG) in the promoter region of clock controlled genes, shutting down their own transcription and decreasing transcription of other genes. Molecular redundancy exists for all core clock genes with three *PER* (*PER1, PER2, PER3*) and two *CRY (CRY1, CRY2)* genes, and paralogues of *BMAL1* and *CLOCK* being *BMAL2* and *NPAS2.* The redundancy likely reflects the importance of circadian clocks to fitness and survival of the animal (Looby and Loudon, 2005). There is also a secondary feedback loop of the core clock, wherein BMAL1 regulates its own transcription by controlling the expression of *REV-ERBA and RORA. RORA and REV-ERBA* encode proteins that compete to bind the retinoid acid receptor response element (RORE) in the promoter region of the *BMAL1* gene. RORA activates while REV-ERBA represses *BMAL1* expression. The periodicity of the core clock transcription-translation feedback loop is about 24 h (Akhtar et al., 2002; Panda, 2002; Storch, 2002). Many of the clock-controlled genes are transcription factors, which results in amplification of the core circadian clock signal (Patton and Hastings, 2018), and so anywhere from 5-10% of genes expressed in a tissue show circadian rhythms of expression. In addition there is circadian oscillation in translation and posttranslational modification of numerous proteins (Chaix et al., 2016), resulting in circadian variation across the scales and into the metabolome of the cell, tissue and across systems.

### Inputs to the master clock in the SCN of the hypothalamus

Light received through the retinohypothalamic tract (RHT) is the primary input to the SCN. Activity, stress, fasting-feeding and nutrients also serve as inputs to the SCN. Moreover, information on metabolic state arrives to the SCN from sympathetic and parasympathetic branches of the autonomic nervous system and hormones and nutrients, such as glucose, that cross the blood-brain barrier (Froy, 2012). Non-photic resetting of the SCN suppresses *PER* gene expression, whereas photic entrainment of the SCN is via induction of *PER* expression (Patton and Hastings, 2018). In addition to changes in core clock genes’ expression, the number of synapses and size and shape of SCN neurons exhibit daily oscillations. This circadian plasticity is important for processing sensory information, learning and memory (Krzeptowski et al., 2018).

Seasonal changes in light drive changes in phase relationship among the core clock genes in the SCN (Porcu et al., 2018). By detecting changes in light level, the clock in the SCN becomes properly phased relative to the external day-night cycle and is therefore attuned to gradual changes in day length (Coomans et al., 2015). In response to seasonal changes in photoperiod the circadian timing system regulates coordinated physiological changes that impact an animal’s growth, energy balance, and reproductive capacity (Lincoln and Richardson, 1998; Clarke et al., 2003; Andersson et al., 2005; Dardente, 2007; Ebling and Barrett, 2008). Neuroplastic changes of the SCN and coordinated changes in pituitary tissue in response to changes in photoperiod length are believed to underlie the generation of circannual rhythms (Hazlerigg and Lincoln, 2011; Wood and Loudon, 2014).

### Outputs of the master clock

The SCN communicates the time of day and photoperiod information to peripheral clocks via output rhythms of hormones and the autonomic nervous system. Neurons that project from the SCN to the spinal column stimulate sympathetic neurons that innervate the pineal gland. This relayed signal results in regulation of melatonin synthesis. Melatonin production occurs in the dark, regardless of rest-activity cycles of the animal. High production of melatonin is maintained in darkness provided there is no light in the environment, as light blocks melatonin production. As an output of the SCN, melatonin in turn functions as an input to all peripheral clocks, and since circulating melatonin varies with length of the dark phase, it reflects seasonal changes in daylength, and acts as a neuroendocrine mediator of the photoperiod (Cipolla-Neto et al., 2014). Circadian rhythms of circulating glucocorticoids are also a primary output of the SCN, in turn glucocorticoids regulate peripheral clocks through multiple mechanisms that include activation of its receptor and binding the glucocorticoid response elements (GRE) present in the promoter regions of *PER1* and *PER2* (So et al., 2009; Lamia et al., 2011; Cheon et al., 2013; Nicolaides et al., 2014). The SCN regulates the circadian release of glucocorticoids via nervous input to the hypothalamo–pituitary–adrenal axis which results in circadian oscillation of adrenocorticotropic hormone (ACTH) and sympathetic innervation of the adrenal gland, which directly controls glucocorticoid release (Lilley et al., 2012). Circadian oscillation of core body temperature is also a primary output of the SCN and functions to influence the timing of peripheral clocks (Buhr et al., 2010). Endocrine rhythms are responsive to factors that compromise the clock function. Nutrients, excess fatness, activity during normal times of rest and stress affect multiple hormones, which feed-back on central and peripheral clocks to adapt circadian rhythms to altered physiological state (Tsang et al., 2014), and vice versa.

### Seasonal rhythms of dairy cows and evidence that the mammary clock is responsive to photoperiod changes

Seasonal, or circannual, rhythms exist as a mechanism for animals to anticipate and adapt to seasonal changes in their environment. Changes in day length (photoperiod) are highly predictable and provide the primary environmental cue of seasonal changes in food availability and climate (Wood and Loudon, 2014). Dairy cattle exhibit seasonal rhythms in hormone secretion and milk production. Seasonal hormone rhythms are typified by prolactin secretion peaking in the summer, and melatonin levels peaking in the winter (Chew et al., 1979; Philo and Reiter, 1980). IGF-1 levels are also higher during long-day photoperiods (Dahl et al., 2000). In the United States, dairy cow milk yield peaks in April and fat and protein yield peak in February. Whereas the circannual nadir in milk yield is in October (Salfer et al., 2019).

Seasonal changes in physiology are regulated at the cellular level by changes in core clocks across the entire animal, with studies showing that changes in photoperiod alter core clock genes expression rhythms in the SCN, pituitary and liver (Andersson et al., 2005; Yasuo et al., 2006; Dardente et al., 2010), as well as the mammary gland (Casey et al., 2018; Kalyesubula et al., 2021). Photoperiod manipulation studies of dairy cows found the impact on milk production is dependent on the production interval the alteration is applied. Long day photoperiod (LDPP; 16 h of light and 8 h of dark) exposure during lactation increases milk yield in dairy cattle without altering feed consumption (Peters et al., 1978). However, when the alteration is applied during the dry period, milk production is increased in the subsequent lactation when cows are exposed to a short-day photoperiod (SDPP) (Dahl, 2008). During the dry non-lactating period, the mammary gland involutes and then is remodeled and redeveloped for the ensuing lactation (Capuco et al., 1997; Capuco and Choudhary, 2020). Data support that SDPP exposure during the dry period increases mammary development resulting in greater milk production in the subsequent lactation (Dahl, 2008). We found that continuing the exposure to SDPP from the dry period into lactation also resulted in higher milk yield in dairy goats relative to LDPP exposed animals (Kalyesubula et al., 2021). The milk production response to photoperiod length during the dry period may more closely reflect the circa-annual rhythms set by the circadian timing system in relation to physiological state of the animal. Adaptations in milk composition and yield to SDPP are speculated to be an evolved means of adequately nourishing calves in seasons with less availability to high-quality forage. The increased milk production due to LDPP exposure only during lactation is believe to be due to increased metabolic activity of tissues (Lincoln and Richardson, 1998).

Multiple investigations have aimed to determine if changes in hormonal milieu reflective of seasonal changes can mimic the photoperiod responses in cattle. Melatonin administration by feeding or implants failed to mimic or only partially mimicked photoperiod responses in production output variables (e.g., Zinn et al., 1988; Sanchez-Barcelo et al., 1991; Lacasse et al., 2014). Thus, although hormonal changes elicit some of the responses, exogenous administration of melatonin fails to capture the integrated and synchronized changes across central and peripheral systems. Part of this failure may be in the approach used for hormone supplementation. Experimental models increased basal hormone levels but did not mimic periodic-rhythmic patterns. Hormone levels normally fluctuate during the day and respond rapidly to cues or stressors (Zavala et al., 2019). High constant stimulation can lead to biological systems becoming refractory to a cue (Han et al., 2007). Periodicity of stimuli is important to biological systems, and maybe particularly important to temporal synchronization of events. Our analysis of the effects of SDPP and LDPP exposures beginning in the dry period of dairy goats found photoperiod differentially affected daily prolactin and temperature rhythms in late gestation and patterns changed with the initiation of lactation (Kalyesubula et al., 2021). Thus, photoperiod interacts with the physiological-reproductive state of the animal to set circadian rhythms. Changes in rhythms and phases of rhythms may be important to timing of events that occur in the mammary gland and other tissues including the liver to prepare for milk production and coordinate production in relation to the animal’s internal and external environment.

Similar to circadian rhythms, circannual rhythms persist even in constant conditions. Thus, the annual rhythms of production are innate to animals. A better understanding of mechanisms that generate seasonal rhythms and responses of tissues to these changes will help in developing novel feeding and reproductive management practices that synchronize with physiological systems of production animals. Current knowledge also needs to be applied to analyzing the effects of changes in feed management on milk production performance within the context of annual rhythms. As an example given by the Harvatine group, 3.6% milk fat may indicate suboptimal milk fat in January but normal milk fat in July. Whereas feeding a dietary supplement in July may appear to improve milk fat percent in the following months. However, the increase in fat may actually be due to the annual rhythm of milk production (Salfer et al., 2019).

## Integration and Reciprocal Regulation Between Circadian and Metabolic Systems

The circadian system coordinates daily patterns of feeding, energy utilization and energy storage across the 24 h day (Gonnissen et al., 2013). Metabolic hormones exhibit circadian rhythms, and the SCN is responsible for the 24 h rhythm in plasma glucose concentrations, with the highest concentrations occurring toward the beginning of the activity period (la Fleur et al., 2001). The hormone ghrelin, which is synthesized in the stomach after feeding, is able to go through the brain-blood barrier and affect the SCN (Carlini et al., 2004). In addition, timing of food intake is an input to circadian clocks in peripheral tissues (Damiola et al., 2000; Stokkan et al., 2001; Eckel-Mahan and Sassone-Corsi, 2013).

At the cellular level circadian clocks interact with nutrient-sensing pathways and nuclear receptors to respond to fasting and feeding states of the animal. Oral administration of short chain fatty acids to mice caused phase changes in the peripheral clocks located in the liver, kidney, and submandibular gland (Tahara et al., 2018). This shifting is due in part to the reciprocal transcriptional regulation between BMAL1 and the nuclear receptor peroxisome proliferator-activated receptor α (PPARA)-transcription factor, which is responsive to fatty acids (Froy, 2012). Additionally, the insulin–pAKT–mTOR pathway interacts with the core clock by way of phosphorylating of casein kinase 1 (CK1) and glycogen synthase kinase 3 (GSK3). CK1 and GSK3 in turn phosphorylate PER, which alters its stability and activity (Zheng and Sehgal, 2010; Eng et al., 2017). Alternatively, during periods of low cellular energy, AMP-activated protein kinase (AMPK) triggers repair and catabolic processes and inhibits mTOR activity (Herzig and Shaw, 2018). AMPK interacts with the clock by phosphorylating CRY to promote its degradation. Another interaction between cellular energy level and the clock is with nicotinamide adenine dinucleotide (NAD+) levels and activity of sirtuins, which vary with the redox state of cells, and affect the activity of the circadian clock by stabilizing BMAL1 chromatin binding (Longo and Panda, 2016; Levine et al., 2020). The synergistic interactions between circadian clocks and feeding-fasting signals coordinate anabolic and catabolic states of metabolism with the animal’s activity–rest cycle. At the systems level this is evident in circadian oscillation of lipid and carbohydrate metabolism coordinated with rhythms of secretion of hormones including insulin, leptin, and cortisol, which induce core clock mediated cellular gene transcription of nutrient transporters and metabolic enzymes at appropriate times, as well as temporal separation of divergent processes, such as glycolysis and gluconeogenesis.

Epidemiological studies of humans have linked circadian disruption with development of diabetes, obesity, and cardiac disease (Karlsson et al., 2001; Lamia et al., 2008; Suwazono et al., 2008). Experimental circadian misalignment in humans blunted leptin rhythms, increased postprandial glucose and insulin, and cortisol rhythms became out of phase with the behavioral rhythm, and half of the participants exhibited a pre-diabetic state during circadian misalignment (Scheer et al., 2009). In reciprocal, excessive fat (obesity) alters circadian rhythms (Kohsaka et al., 2007; Mendoza et al., 2008). Rhythmic gene expression is attenuated in mice with genetically induced obesity (Ando et al., 2005). Mice with diet induced obesity exhibit a delay in circadian entrainment to light-phase shift (Mendoza et al., 2008). The effect of high fat diet on clock function is very rapid and occurs before development of obesity. Changes are observed in behavior and rhythms of the liver clock occur within three days of starting a high fat diet (Eckel-Mahan et al., 2013). In the obese state, levels of glucose and insulin were found elevated in rats throughout the day, and growth hormone, prolactin and thyroxine were depressed. Whereas circadian rhythms of circulating corticosterone were attenuated and levels elevated throughout the circadian cycle in diet induced obese rats (Martin et al., 1978). Similarly, in obese humans, basal levels of cortisol are higher with an attenuation of the daily rhythm (Pasquali et al., 2006) and a lengthening of rhythm period (Bass and Takahashi, 2010).

### Studies in cattle and evidence that the mammary clock is entrained to feeding time

Eating, rumination, and rumen pH of dairy cattle show exhibit daily rhythms (DeVries et al., 2003; Salfer et al., 2018). Multiple metabolic hormones including insulin, somatotropin, cortisol, melatonin, and triiodothyronine show circadian rhythms of secretion in dairy cows (Hedlund et al., 1977a, 1977b; Bitman et al., 1994; Lefcourt et al., 1993, 1994, 1995, 1999). Blood metabolites such as glucose, nonesterified fatty acids (NEFA), β-hydroxybutyrate (BHBA), and urea nitrogen also exhibit daily rhythms in cows (Bitman et al., 1990; Lefcourt et al., 1999). Non-pregnant, non-lactating dairy cows exhibit circadian oscillations of locomotor activity, rectal temperature, respiratory rate, hemoglobin, glucose, creatinine, urea, total cholesterol, total lipids, non-esterified fatty acid (NEFA), phosphorus and magnesium (Giannetto and Piccione, 2009a, 2009b). Studies of sheep also demonstrated robust daily rhythms in reactive oxygen metabolites indicating that circadian system likely maintains the balance between production and removal (Piccione et al., 2010).

Restricted feeding induces food anticipatory activity which is characterized by an increase of locomotor activity, a rise in core body temperature, and elevated serum corticosterone in rodents. Ruminants also show food anticipatory activity, as circadian rhythms of activity and temperature similarly shifted in response to timed meal feeding in sheep and goats (Piccione et al., 2003, 2007; Giannetto et al., 2010). Our studies of dairy cows found evidence for a food anticipatory rise in core body temperature at three weeks before expected calving in non-lactating (dry) cows (Suarez-Trujillo et al., 2022). In lactating cows, feeding time affects circadian rhythms of feeding and lying behavior, and core body temperature (Niu et al., 2014). The highly predictable nature of shifts in rhythms that develop with food anticipatory activity to timed feeding support a centrally regulated food entrainable oscillator outside of the SCN, which stays locked to the light–dark cycle. Current research suggests that the dopaminergic nuclei in the midbrain may be a candidate (de Lartigue and McDougle, 2019).

Studies of rodents have clearly shown that phases of peripheral clocks in the liver, pancreas, kidney, and muscle are shifted by feeding time (Damiola et al., 2000). When feeding time is restricted to typical times of rest, it can be completely dissociated from the SCN, demonstrating that the time of food availability is a stronger input cue for peripheral clocks than the light–dark cycle. Studies conducted by the Harvatine lab support that timing of food intake is an input to the mammary clock. Restricting feeding times in lactating mice caused shifts in mammary clocks genes’ expression and circadian variations of milk fat synthesis by affecting lipogenic regulators and milk fat synthetic enzymes (Ma et al., 2013). Milk yield and milk fat and protein concentration exhibit circadian rhythms in dairy cows (Rottman et al., 2014), and restricting feed intake of dairy cows to the night versus the day shifts core body temperature, plasma metabolites and milk production (Salfer and Harvatine, 2020). Mammary core clock genes’ expression was also shifted between day and night restricted feeding which was accompanied by changes in the timing of de novo fatty acid synthesis in the gland (Salfer and Harvatine, 2020). In support of a role of mammary clock in regulating fatty acid synthesis and metabolism, our ChIP-seq analysis of transcriptional targets of BMAL1 in mouse mammary epithelial cells identified genes that regulate uptake, transport and synthesis of lipids (Casey et al., 2021). Moreover, genes that regulate fatty acid synthesis were shown to exhibit circadian rhythms of expression in lactating mammary tissue (Maningat et al., 2009), and our studies of mid-lactation cows found circadian disruption due to exposure to continuous light-dark phase shifts decreased expression of *fatty acid synthase (FASN*) and *acetyl CoA-carboxylase (ACACA)* in the mammary gland (Casey et al., 2014a).

### Impact of circadian disruption on metabolism in late pregnant dairy cattle

In humans, exposure to circadian disruption, such as with factory or hospital shift work, increases the risk of obesity, high blood pressure, hyperglycemia, insulin resistance, non-alcoholic liver disease and heart disease (Golombek et al. 2013). The effects of circadian disruption can be exacerbated in pregnant women with increased risk for gestational diabetes and maternal and neonate morbidity (Aylamazyan et al. 2018). Our studies of late gestation dairy cows found circadian disruption induced by exposure to continuous shifts in light-dark cycles (a chronic jet-lag paradigm) in late gestation resulted in cows developing hypoglycemia which carried over into early lactation (Suarez-Trujillo et al. 2020). In a follow-up study, cows exposed to light-dark phase shifting were found to have decreased insulin sensitivity in response to intravenous glucose tolerance test (IVGTT) (McCabe et al. 2021b). Similarly, studies of pregnant sheep found exposure to circadian disruption using a chronic shift work model (alternating light–dark patterns using 12 h shifts) resulted in hypoglycemia, and in a second study decreased insulin sensitivity in response to IVGTT (Varcoe et al., 2014; Gatford et al., 2019).

To further our understanding of what may be leading to altered glucose homeostasis, we analyzed carbon flux in hepatic tissue biopsied from late gestation cows. Liver incubated with uniformly labeled ^13^C-propionate showed lower flux toward gluconeogenesis in cows exposed to continuous phase-shifting of light-dark cycles relative to controls (manuscript in review). Analysis of genes related to changes in carbon flux ratios indicated that propionate was preferentially used for energy generation rather than gluconeogenesis in cows with disrupted circadian rhythms. Rodents exposed to circadian disrupting environments also exhibited a decreased capacity for gluconeogenesis (Lamia et al., 2008; Marcheva et al., 2010). Ruminants rely on gluconeogenesis for 90% of their glucose supply (Young, 1977), and thus minimizing exposures to factors that disrupt circadian clocks may be particularly important in late gestation and early lactation cows, when glucose requirements are particularly high (Bell and Bauman, 1997). Limiting exposure to chronodisruptors may also decrease the risk for developing diseases in the peripartum period, as global analysis of the impact of exposing late gestation dairy cows to circadian disruption on hepatic transcriptome and muscle proteome found a potential for an increased risk of developing fatty liver (Casey et al. 2021), and increased oxidative stress in muscle tissue (McCabe et al., 2021a).

Circadian clocks are a central component of an animal’s homeostatic system, chronic disruption of clocks negatively impacts metabolic systems. Light, stress, exercise, and nutrition are inputs to the circadian timing system. Management systems that disrupt 24 h cycles of these inputs can disrupt circadian clocks, and potentially affect the health, welfare and production efficiency of dairy cattle. Managing cows to limit exposure to chronodisruption may better help them adapt during times when coordinated changes in metabolism must occur to support fetal growth and milk synthesis. Developing management and feeding systems that meet nutrient needs while synchronizing and coordinating compatible temporal events in metabolism with other rhythms of behavior and physiology may lead to more robust rhythms (Kuhlman and McMahon, 2006). Whether temporally restricting feeding and coordinating feeding with other physiological rhythms can have beneficial effects on production efficiency in cattle needs to be determined.

## Circadian System Regulation of Reproduction

Similar to the circadian and metabolic systems of the body, the reproductive and circadian systems are integrated and reciprocally regulated. Reciprocal regulation and interaction among circadian, metabolic and reproductive systems is most evident in seasonal breeders, for which reproductive cycles are regulated to time the birth of young when food is most available (Lincoln and Richardson, 1998; Harrison et al., 2008; Barrett and Bolborea, 2012). Although cows are not seasonal breeders, season influences the establishment of estrous cycles at puberty and after calving with potentially an increased likelihood of spring calving (Hansen, 1985). Beyond the synchronization of reproductive events to the time of year, circadian clocks play a central role in regulating the precise timing of hormone release and other events along the hypothalamal–pituitary–ovarian axis to optimize fertility (Sen and Hoffmann, 2020).

### Circadian clock regulation of reproductive timing

Successful reproduction in females is dependent on coordinating the timing of ovulation with a receptive uterus. SCN lesion studies of rodents found loss of the master clock resulted in infertility due to the lack of the ability to synchronize events for ovulation (Turek et al., 1994). Genetic disruption of circadian clocks also decreases fertility of mice (Dolatshad et al., 2006; Liu et al., 2014; Tonsfeldt et al., 2019). Infertility of the *Bmal1* knockout mice is believed to be due in part to defects in progesterone synthesis in the ovary which results in implantation failure (Tonsfeldt et al., 2019). Moreover, although the *Bmal1* knockouts ovulate (although infrequently), there is no detectable luteinizing hormone (LH) surge (Chu et al., 2013). In wild-type mice, the preovulatory LH surge that stimulates ovulation takes place approximately every 4 to 5 d at the end of the resting period (i.e. at the late day–early night transition in nocturnal rodents). When mice were exposed to a single 10-h phase advance or delay in the start of the light cycle (acute circadian disruption), they continued to display estrous cycles but the length of the cycle was longer and it took 3 cycles for the LH surge to return (Bahougne et al., 2020). When mice were exposed to chronic light–dark phase shifts, the LH surge was impaired and resulted in half the number of pups of control mice.

In humans, circadian disruption has been linked to reproductive dysfunction and subfertility (Sen and Sellix, 2016). Women report menstrual cycle irregularities at higher rates when in chronodisruptive environments, such as shiftwork (Baker and Driver, 2007). Chronic circadian disruption in humans is related to an increased latency to pregnancy, and miscarriages, with the greatest risk in the first trimester. Chronic shift work is also associated with increased risk of preterm birth and low birth weight infants (Sen and Sellix, 2016). Although it has not been possible to establish a causal-link to clock function in any single tissue of the HPG axis in humans, these data strongly indicate circadian disruption affects fertility. Moreover, as discussed above circadian disruption has a confounding influence by causing metabolic dysfunction. Insulin resistance, dyslipidemia, and obesity are all linked to decreased fertility in humans (Rasmussen and Kjolhede, 2008).

Studies of the impact of circadian disruption on ruminant reproduction are limited, but existing data suggest that exposure to chronodisruptors decrease reproductive competence. The endocrine milieu of late pregnant ewes and their fetuses was significantly altered when transitioned to constant light for 48 h compared to control groups that remained on 12 h light-12 h dark cycles. The constant light increased levels of follicle-stimulating hormone and estradiol and decreased progesterone levels in both maternal and fetal circulation (Gao et al., 2016). Low levels of melatonin are linked with adverse pregnancy outcomes, and supplementing pregnant ewes with melatonin was shown to increase arterial blood flow in the placenta (Shukla et al., 2014). Melatonin is an important antioxidant that scavenges free radicals which can impair fertility (Weiss et al., 2009), and so lighting conditions that limit its synthesis may diminish the likelihood of a successful pregnancy. Dairy cows are known for low reproductive efficiency. Reproductive efficiency of cattle has been associated with management practices, housing, and milk production (Ferguson and Skidmore, 2013). It is unclear at this time whether chronodisruption due to light–dark cycles in production facilities or exposure to other disruptors may be playing a role, but it is quite possible that limiting chronodisruptors may help in management of the fertility issue of dairy cows.

Circadian clocks are also important to timing parturition. In mammals, labor preferentially occurs during the rest phase of the day, which translates into labor at night in diurnal species (Bosc, 1990). The mechanisms timing labor to the rest phase of the day varies dependent on the species, but include signals from the fetus (Mesiano and Jaffe, 1997), the maternal and fetal SCN (Reppert et al., 1987) and the uterus (Ratajczak, Asada et al. 2012). In primates, the nocturnal onset of labor coincides with increased sensitivity of the pregnant uterus to oxytocin and melatonin, two hormones driving pregnancy associated uterine contractions (Hirst et al., 1993; Olcese et al., 2013; Olcese 2014). Studies of sheep have shown that circadian disruption during pregnancy increased gestation length (Gatford et al., 2019), and we similarly found longer gestation length in cows exposed to chronic light-dark phase shifts during the dry period (Suarez-Trujillo et al., 2022). In this study we observed that cows exposed to regular 24 h light-dark cycles (control) exhibited more robust circadian rhythms of core body temperature, cortisol, and serotonin as they approached parturition. We hypothesized that changes in rhythms reflected changes in underlying clocks that function to coordinate the timing of parturition, and potentially control the coordinated metabolic changes that occur across multiple tissues, which is needed to support milk synthesis during lactation.

### Changes in circadian system related to reproductive state

Major changes occur in the physiology and behavior of females at the onset of pregnancy and lactation (Bauman et al., 1989; Bell and Bauman, 1997; Bauman, 2010). Among the adaptations to these physiological states are changes in circadian rhythms of behavior and physiology. There is a general dampening of daily rhythms of activity in rodents beginning in late gestation and a substantial disruption at parturition. Rhythms of activity remain disrupted through peak lactation in rat dams, and start to return in the last third of lactation when neonates begin to wean naturally (Scribner and Wynne-Edwards, 1994). Similarly, in humans, the day-to-day stability of rest-activity rhythms diminishes throughout pregnancy and becomes completely disordered in the early postpartum period (Nishihara et al., 2012; Krawczak et al., 2016; Casey et al., 2020). Circadian rhythms of core body temperature and circulating glucocorticoids also change substantially with the onset of pregnancy and into early lactation. Circadian rhythms of core body temperature are dampened (attenuated) in gestating rats relative to nonpregnant controls, with higher basal temperature across the day (Schrader et al., 2009). At the onset of lactation basal core body temperature increases in rats and there is a further decrement in rhythmicity. The attenuation of core body temperature rhythms is evident through peak lactation (day 10), and similar to the return of robust circadian rhythms of activity, there is a return to strong circadian rhythms of core body temperature in late lactation (day 19) when rat pups are naturally weaning. The subparaventricular zone (SPZ) of the hypothalamus is the main efferent target of the SCN, and functions to relay information to the peripheral system through generation circadian rhythms of core body temperature and activity (Vujovic et al., 2015). Relative to the non-pregnant state, the SCN and vSPZ were found reorganized in early pregnant rodents, especially in the way they responded to photic cues (Schradera et al., 2010), and thus likely contribute to changes in behavioral and physiological rhythms that occur in females with the initiation of pregnancy.

Circadian rhythms of hormones also become attenuated during lactation. Basal prolactin levels are elevated in rats during peak lactation, and the circadian rhythm of prolactin observed during estrous cycles is lost in lactating animals. Diurnal variations in ACTH and corticosterone remain in lactating rats, however relative to virgin animals, rhythms are attenuated with elevation in trough levels (Walker et al., 1992). Lactating rats also exhibited a lower release of ACTH and corticosterone in response to stress. Similarly in humans, the circadian rhythm of saliva cortisol is maintained throughout pregnancy, despite a progressive decline in the cortisol awakening response and maternal stress reactivity (Obel et al., 2005; Entringer et al., 2010).

Changes in circadian rhythms of activity, temperature and hormones have also been observed in ruminants as they transition across reproductive states. Nonpregnant, non-lactating cows exhibit circadian rhythms of activity, with greatest activity during the light phase of a light–dark cycle. At the onset of lactation, cow activity becomes significantly diminished with daily patterns of activity influenced by milking and feeding times (Piccione et al., 2011). During the transition from late gestation to early lactation dairy cows (Suarez-Trujillo et al., 2022) and goats (Kalyesubula et al., 2021) exhibited diminished temporal organization of daily rhythms of body temperature and multiple hormones. The major physiological changes that occur in dairy cows at parturition with the initiation of lactation are accompanied by major changes in management that include housing, social interactions, initiation of milking and feed. These changes likely contribute to the loss of the daily rhythmicity in early lactation dairy cows. An observational study we conducted of early lactation dairy cows aimed to determine if there was a relationship between activities of cows in a free-stall with daily variations in their body temperature (manuscript in preparation). There was no relationship between eating behavior and body temperature, however there was a significant effect of milking time on temperature increment. Two component cosinor analysis indicated two peaks of temperature within a 24-h period coincident with milking time. Others have reported similar biphasic rhythms of core body temperature in cows milked twice daily (Kendall et al., 2008). Milking time encompasses a period of increased activity, social interactions, and interactions with humans, and milking in the parlor. Exercise and stress are associated with increased body temperature (Arave et al., 1987). Milking may also increase body temperature. Milking stimulates a neuroendocrine response that results in the release of oxytocin, prolactin, and glucocorticoids (Mattheij et al., 1979; Barofsky et al., 1983; Fuchs et al., 1984; Deis et al., 1989; Pi and Voogt, 2001; Bodnar et al., 2004; Brunton et al., 2008). Oxytocin is thermoregulatory, and exogenous administration of oxytocin to rodents increased core body temperature (Camerino, 2021). Further research in this area is needed as understanding factors that affect body temperature oscillations and their regulation need to be considered in the design of systems aimed at mitigating heat stress effects on milk production and dairy cow welfare. Consideration of regulation by circadian clocks may be particularly important. Genome-wide association study (GWAS) of cattle, transcriptome studies of rats and phenome-wide association analysis of human data identified five genes as being associated with heat stress response across mammal species, two of which—*ARNTL* (a.k.a. *BMAL1*) and *NPAS2* (*CLOCK*’s paralogue)—are core clock genes (Dou et al., 2021). Moreover, an understanding of changes in daily activities in relation reproductive events and disease in cows may help in developing good management systems in large production facilities. Recent mathematical analysis of continuously monitored daily rhythms of behavior of dairy cows was able to identify cows with diseases or reproductive events by disrupted rhythms (Wagner et al., 2021).

During pregnancy and lactation, the survival of offspring becomes a priority. Cues emanating from the conceptus during pregnancy and the neonate during lactation affect maternal behavior and physiology. The changes in maternal behavior that occur during gestation and lactation are due, in part, to changes in endocrine milieu associated with reproductive states and nursing demands of neonates (Prendergast et al., 2012). Changes in the dam occur across all scales of the animal, from gene expression to systems level and include tissue specific changes in core clock dynamics. In late gestation rats, core clock gene expression rhythms were found diminished in placenta compared with relatively robust rhythms of core clock gene expression in liver tissue (Wharfe et al., 2011). Recent studies of explants of bovine placenta at 180 d of gestation demonstrated a similar attenuation of core clock genes’ expression rhythms, whereas mRNA expression of *HIF1alpha* and the glucocorticoid receptor showed distinct 24-rhythms (Contreras-Correa et al., 2020). The attenuation of clock genes’ expression rhythms in the placenta is speculated to ensure 24 h activation of downstream genes that are needed to supply nutrients to growing fetus. Our studies of mice compared core clock genes’ expression rhythms in SCN, liver and mammary tissue of late pregnant and early lactation mice (Casey et al., 2014b). The robustness of core clock genes’ expression rhythms increased in hepatic tissue and the SCN from pregnancy to lactation, and were speculated to compensate for the increased metabolic demands. However, in the mammary gland there was an attenuation of circadian rhythms of expression of core clock genes and appearance of an ultradian pattern of expression of *BMAL1.* The attenuation of core clock expression rhythms in mammary tissue during lactation led us to hypothesize that clocks were responsive to metabolic cues initiated by neonate during suckling. Our cell culture studies showed that core clock genes’ expression patterns were shifted in response to the addition of prolactin and glucocorticoids to cultures. This finding supports the hypothesis that the mammary clock is responsive to input cues initiated by the neonate by suckling, or milking as in dairy production species. This makes sense, in light of findings of photoperiod studies that found differences in milk yield. Moreover, temporal analysis of mammary epithelial gene expression during human lactation found 7% of the genes exhibited circadian oscillation in expression, and these genes regulated processes important for milk synthesis (Maningat et al., 2009). In mice, mammary expression of *LALBA* (alpha-lactalbumin), *SREBF1* (sterol regulatory-element-binding protein 1), and *FASN* (fatty acid synthase) genes all showed circadian rhythms during lactation (Casey et al., 2014b). The circadian rhythm of lactose synthesis is well characterized and known to be mediated by circadian changes of expression in lactose synthesis enzymes (Kuhn et al., 1980). Studies of lactating sheep revealed *BMAL1* and *PER2* showed circadian patterns of expression in RNA isolated from milk fat globules in ewe’s milk which correlated with circadian changes in expression of *acetyl-CoA carboxylase* (*ACACA*) as well as percent milk fat (Schmitt et al., 2014). Thus, the attenuation in maternal behavior, hormones, core body temperature and core clock genes’ expression in the mammary and changes in hepatic rhythms demonstrate the flexibility of the maternal circadian system to respond to the metabolic demands of lactation, and timing of the demands established by suckling of neonate or milking initiated cues as in dairy production animals.

### Impact of circadian disruption on mammary development and lactation

Studies of circadian regulation of reproduction in the *Clock-Δ19* line of mice found the mutation had minimal effects on growth and development of litters during gestation, however pup growth and survival were significantly decreased postnatal (Kennaway DJ, 2004; Dolatshad et al., 2006; Hoshino et al., 2006). Death was not due to neonate genotype, indicating that maternal phenotype was affecting postnatal litter survival. Basal serum prolactin levels were not different between mutant and wild-type dams; however, *Clock-Δ19* mice exhibited altered nursing behavior that was marked by increased frequency and longer bouts (Hoshino et al., 2006), which is an indicator of impaired milk production. Our analysis of *Clock-Δ19* mice found a high rate of pup mortality postnatal that was related to poor mammary development in late gestation (Casey et al., 2016). Studies of cattle, also indicate a central role for circadian clocks in regulating mammary development during lactation. Disrupting circadian clocks of dairy cows during late gestation by exposing them to chronic light–dark phase shifts decreased mammary epithelial proliferation and ratio of lumen to epithelial area, which was related to lower milk production in the subsequent lactation (McCabe et al., 2021b). Milk yield was also reduced in mid-lactation cows when they were exposed to chronodisrupting light–dark phase shifts (Casey et al., 2014a). Our studies of women found lower sleep efficiency and night-to-night variation in sleep, which disrupt circadian clocks, was related to a higher risk for delayed lactogenesis II (secretory activation) (Casey et al., 2019). ChIP-seq analysis of transcriptional targets of BMAL1 support a role for the mammary epithelial clock in regulation of genes that regulate milk synthesis, growth and differentiation of epithelial cells as well as the coordination of hormonal signals with nutrient uptake, which is needed to initiate lactation (Casey et al., 2021). Moreover, in 2D cultures of *BMAL1* knock-out cells there was an increased rate of cell death related increased levels of reactive oxygen species and lower expression of super-oxide dismutase 3 (SOD3). Deletion of *BMAL1* in mammary epithelial cells led to decreased ability to form acini in 3D cultures. Together indicating multiple ways in which circadian clock disruption has the potential to impair mammary development and the signaling needed to initiate the timing of the onset of lactogenesis, milk synthesis, and maintenance of tissue homeostasis, which is important to lactation persistence.

## Concluding Remarks

The circadian system enables animals to follow time. This ability is important for the temporal separation of incompatible processes and the maximal efficiency of others. There is a growing understanding of factors that affect circadian clock function. These factors include light, season, nutrition, and stress as well as reproductive status of the animal. Although more work needs to be done, current dairy systems should consider these factors in determining how they impact production efficiency and welfare of cattle. More knowledge and application of this knowledge may lead to better scheduling of daily management practices that are consistent with the cows’ physiology and behavior, as well as interpretation of production data in relation to the time of year. Knowledge of daily patterns of cow behavior and physiology can help in the identification of disturbances in the system that may be related to reproductive events or indicative of disease. Limiting exposure to factors that disrupt circadian clocks, especially when cows are metabolically challenged may be particularly important to minimizing the risk for disease, it may also potentially increase reproductive efficiency.

## Abbreviations

ACTH: adrenocorticotropic hormone
AMPK: AMP-activated protein kinase
CK1 GSK3: casein kinase 1 and glycogen synthase kinase 3
CRY: cryptochrome
E-box: enhancer box
GRE: glucocorticoid response elements
HPG: hypothalamal–pituitary–gonadal axis
LDPP: long day photoperiod
LH: luteinizing hormone; NAD+. nicotinamide adenine dinucleotide
NEFA: nonesterified fatty acids
BHBA: β-hydroxybutyrate
PER: period
RHT: retinohypothalamic tract
RORE: receptor response element
SCN: suprachiasmatic nuclei
SDPP: short-day photoperiod
SPZ: subparaventricular zone
